# Copper–molybdenum interactions in ruminant nutrition and reproductive physiology: mechanistic insights into growth, digestibility, and follicular development

**DOI:** 10.3389/fvets.2026.1878484

**Published:** 2026-09-30

**Authors:** Junfang Hao, Qian Xu, Kai Huang, Jiangbo Zhang

**Affiliations:** 1 College of Biology and Food, College of Smart Animal Husbandry, Shangqiu Normal University, Shangqiu, Henan, China; 2 College of Veterinary Medicine, Northwest A&F University, Yangling, Shaanxi, China; 3 Zhoukou Municipal Product Quality Inspection and Testing Center, Zhoukou, Henan, China; 4 Agricultural and Rural Service Center of Longxiang Sub-district Office, Zhongzhan District, Jiaozuo, Henan, China; 5 College of Animal Science and Technology, Xinyang Agricultural and Forestry University, Xinyang, Henan, China

**Keywords:** copper, digestibility, follicle characteristics, molybdenum, ruminant growth

## Abstract

The copper–molybdenum–sulfur (Cu–Mo–S) interaction is one of the most important mineral antagonisms in ruminant nutrition because it can induce functional copper deficiency even when dietary copper intake appears adequate. In the rumen, dietary sulfur is reduced to sulfide, which reacts with molybdate to form thiomolybdates. These compounds bind copper within ruminal digesta and, when produced in excess, may also enter the circulation and interfere with systemic copper trafficking and cuproenzyme function. This review critically synthesizes evidence on ruminal thiomolybdate formation, post-absorptive copper sequestration, growth and immune consequences, rumen fermentation, and hair/wool follicle abnormalities. Practical mitigation requires more than increasing dietary copper: feed, forage, mineral supplements, and drinking water should be analyzed together for Cu, Mo, S, Fe, and Zn; dietary Cu:Mo:S balance should be interpreted in a species-specific manner; high Mo and sulfur exposure should be reduced when possible; and copper supplementation should be adjusted cautiously, particularly in sheep because of their narrow margin between deficiency and toxicity. Rumen-stable or less rumen-soluble copper sources, such as hydroxychloride or selected organic copper complexes, may reduce ruminal antagonism under specific feeding conditions, although responses remain diet- and species-dependent. Diagnostic interpretation should combine serum copper, liver copper, ceruloplasmin or SOD-related indices, and, where available, functional biomarkers such as CCS or TCA-fractionated plasma copper. The review concludes that effective management of Cu–Mo–S imbalance requires an integrated framework linking diet and water testing, functional copper-status assessment, species-specific risk thresholds, and targeted supplementation rather than broad group-average mineral feeding.

## Introduction

The biological indispensability of copper is a fundamental tenet of ruminant physiology, as this trace element serves as a mandatory cofactor for a diverse array of cuproenzymes that regulate cellular respiration, antioxidant defense, connective tissue integrity, and iron homeostasis (1, 2). Despite its essentiality, the bioavailability of copper is precarious in the ruminant due to the unique anaerobic environment of the rumen, which serves as a staging ground for a potent antagonistic interaction involving molybdenum and sulfur (3, 4). This relationship, characterized by the formation of thiomolybdates, represents a paradigm of “conditioned” deficiency where clinical signs of hypocuprosis often occur despite seemingly adequate dietary copper levels. The historical recognition of this problem—dating back to early observations of “salt-sick” cattle and “swayback” lambs—has evolved into a sophisticated field of nutritional biochemistry that now seeks to resolve the ambiguities between secondary copper deficiency and direct thiomolybdate toxicity. The economic impact of these mineral imbalances is profound, extending from subclinical reductions in average daily gain and feed efficiency to the total loss of marketable fiber quality and increased mortality due to immunosuppression (5, 6). In the modern production landscape, the challenge is further complicated by the narrow margin between deficiency and toxicity, particularly in sheep (7), which possess a limited capacity for biliary copper excretion and are thus highly susceptible to chronic copper poisoning if molybdenum levels are insufficient. As we transition into an era defined by precision agriculture and high-throughput diagnostic tools, it is imperative to re-evaluate our understanding of the Cu-Mo-S triad. This involves not only a deeper investigation into the ruminal mechanisms of mineral sequestration but also an assessment of how these interactions influence systemic metabolic pathways, from the oxidative status of the pancreas to the genomic regulation of hair and wool follicles (8–10). The current state of the industry reveals a significant gap between laboratory insights and field applications, particularly regarding the use of molecular biomarkers and individualized supplementation strategies. This review aims to bridge that gap by providing a high-level scientific evaluation of the current insights regarding the influence of copper and molybdenum on ruminant growth, digestibility, and follicle characteristics. By synthesizing data from recent PubMed-indexed studies and emerging multi-omics research, the following analysis delineates the pathways through which these minerals dictate the productive lifespan of the animal and proposes a roadmap for future directions in precision mineral management, genomic selection, and epigenetic programming. Given the substantial heterogeneity among available studies in species, breed, dietary Cu:Mo:S ratios, sulfur source, basal copper status, exposure duration, and measured outcomes, the evidence is synthesized qualitatively, with quantitative values reported where available.

## The biochemical architecture of the ruminal Cu-Mo-S interaction

The rumen acts as a complex bioreactor where the bioavailability of trace minerals is dictated by microbial activity and chemical shifts (11). The primary mechanism of the copper-molybdenum-sulfur interaction is the synthesis of thiomolybdates, a family of oxythiomolybdates formed when the oxygen atoms of the molybdate ion Mo4O2 are progressively replaced by sulfur atoms derived from ruminal sulfide (12–14). This process is highly dependent on the presence of microorganisms capable of reducing inorganic sulfate or degrading sulfur-containing amino acids into hydrogen sulfide (H2S) (15, 16). Recent rumen microbiome and fermentation studies have further emphasized that sulfur metabolism should not be treated only as a chemical input but also as a microbial functional trait (17, 18). Sulfate-reducing bacteria can use hydrogen as an electron donor and reduce sulfate to sulfide/H₂S, particularly when ruminal sulfur availability is increased or fermentation conditions are altered (17, 19). This increased sulfide availability provides the biochemical substrate required for thiomolybdate formation, as described in the classical Cu–Mo–S model (12–14). However, direct metagenomic identification of thiomolybdate-forming microbial consortia remains limited, and future studies should integrate rumen metagenomics, sulfur-metabolism gene profiling, and direct thiomolybdate quantification (17, 18).

The chemical progression follows a stepwise substitution:

1. 1 Molybdate (MoO_4_^2−^)
2. 2 Monothiomolybdate (MoO_3_S^2−^)
3. 3 Dithiomolybdate (MoO_2_S_2_^2−^)
4. 4 Trithiomolybdate (MoOS_3_^2−^)
5. 5 Tetrathiomolybdate (MoS_4_^2−^)

Each subsequent substitution increases the affinity of the molecule for copper (12). Tetrathiomolybdate (TTM) is recognized as the most potent antagonist, as it forms highly stable, insoluble complexes with copper (CuMoS4) within the rumen contents (12, 20). These complexes are too large or chemically inert to be absorbed in the small intestine, leading to their excretion in the feces and a resultant decrease in the net copper status of the animal (12, 21) (Table 1).

**Table 1:** Different compounds of molybdenum in combination with copper and sulfur.

Step	Compound name	Formula	Potency as Cu antagonist	Ref
1	Molybdate	MoO_4_^2−^	Negligible	(12)
2	Monothiomolybdate	MoO_3_S^2^	Low	(12)
3	Dithiomolybdate	MoO_2_S_2_^2^	Moderate	(12)
4	Trithiomolybdate	MoOS_3_^2^	High	(12)
5	Tetrathiomolybdate	MoS_4_^2^	Very high	(12)

Crucially, the interaction does not end in the rumen. If the production of thiomolybdates exceeds the amount of rumen-available copper, these compounds are absorbed into the systemic circulation (12). Once in the blood, thiomolybdates bind to copper already associated with plasma albumin and can even sequester copper from metalloenzymes, rendering it metabolically inactive (12, 22). This creates a paradoxical diagnostic scenario where the animal may exhibit clinical signs of copper deficiency while having “normal” or even elevated total plasma copper levels, as the copper is “locked” in a TCA-insoluble thiomolybdate–copper–protein complex (12, 22). The use of trichloroacetic acid (TCA) to fractionate plasma copper is essential; copper found in the TCA-precipitate (insoluble fraction) is a direct indicator of systemic thiomolybdate exposure (12, 22).

## Critical synthesis of ruminal and post-absorptive mechanisms of copper antagonism

The Cu–Mo–S interaction should be interpreted as a sequence of linked ruminal and systemic events rather than as a simple reduction in dietary copper availability. In the rumen, the extent of antagonism depends on the simultaneous availability of soluble molybdate, reducible sulfur, sulfide production, and rumen-soluble copper. When rumen-soluble copper is available, thiomolybdates are more likely to form insoluble Cu–Mo–S complexes within digesta and be excreted. When thiomolybdate production exceeds rumen-available copper, however, thiomolybdates may be absorbed and bind systemic copper pools, thereby reducing functional copper availability despite apparently adequate or even misleadingly normal total plasma copper.

This explains why dietary Cu concentration alone is an incomplete predictor of animal copper status. Dietary sulfur source and concentration, molybdenum source, water mineral composition, iron and zinc antagonism, forage type, rumen pH, and trace-mineral source can all modify the effective Cu–Mo–S challenge. For example, sulfate trace-mineral sources may increase ruminally soluble mineral pools and alter copper binding to digesta, whereas hydroxychloride sources appear less soluble in the rumen and may be less tightly bound to ruminal solid digesta (19, 23). Therefore, the practical question is not simply whether a diet contains “enough copper,” but whether the animal receives enough metabolically available copper after ruminal sequestration and systemic thiomolybdate interference.

Current evidence also indicates several unresolved mechanisms. First, the microbial taxa and functional genes directly responsible for sulfur reduction and sulfide availability under high-Mo diets remain insufficiently characterized. Second, species differences in copper storage, biliary excretion, and tolerance to copper accumulation complicate the extrapolation of cattle data to sheep or goats. Third, conventional indicators such as serum copper and liver copper do not always distinguish primary dietary copper deficiency from thiomolybdate-induced functional copper blockade. These gaps support the need for integrated diet-water mineral analysis, functional copper-status biomarkers, and controlled studies linking rumen chemistry, microbial sulfur metabolism, thiomolybdate speciation, and tissue-level copper-dependent enzyme activity.

## Influence on growth performance and metabolic development

The impact of copper-molybdenum imbalances on growth performance is a multi-faceted issue that varies by ruminant species, age, and the duration of exposure. While clinical deficiency is characterized by visible wasting, the subclinical “ill-thrift” caused by these minerals is far more common and economically damaging due to its surreptitious nature (24). A previous meta-analysis in growing-finishing cattle also reported that dietary Mo and S were negatively associated with average daily gain and feed conversion, while the dietary Cu: Mo ratio showed linear and quadratic associations with ADG (25).

## Species-specific growth responses in goats and sheep

Research in Boer-cross goats has demonstrated that low-copper diets supplemented with increasing molybdenum (5 ppm and 10 ppm) do not necessarily result in a significant depression of average daily gain (ADG) or final body weight over an 85-day period (24). This suggests that goats may possess a degree of resilience, possibly by mobilizing hepatic copper stores more effectively than other species or through differences in ruminal passage rates (24). However, even in the absence of a weight gain deficit, these goats showed a linear decrease in liver copper and iron concentrations, indicating that the animal’s internal reserves were being depleted to maintain outward performance (24, 26). In contrast, sheep are significantly more vulnerable. High molybdenum intake in sheep has been shown to reduce both weight gain and the efficiency of food conversion (12, 27). Because sheep are also uniquely susceptible to copper toxicity, the balance must be managed with extreme precision (28). A Cu: Mo ratio of less than 4.5:1 is often cited as the threshold for inducing deficiency, whereas a ratio exceeding 10:1 significantly increases the risk of chronic copper poisoning (28, 29). In growing-finishing cattle, a previous meta-analysis showed that dietary Cu, Mo, and S and their interactions are associated with plasma and liver Cu status, average daily gain, and gain-to-feed ratio (25).

## Cattle and long-term feedlot performance

In beef cattle, the interaction is often managed by fortifying diets with trace minerals above NASEM requirements to account for antagonistic pressures from molybdenum and sulfur in forage and water (29, 30). Studies on steers consuming high-concentrate diets have shown that while the source of the mineral (sulfate vs. hydroxychloride) may not drastically alter overall nutrient digestibility, it can significantly impact the ruminal solubility of copper and the strength of its binding to digesta (19, 23). Furthermore, steers receiving supplemental molybdenum and sulfur may show a reduction in plasma copper over extended exposure periods, eventually leading to impaired growth if mineral imbalance is not corrected (24, 31). Species-specific responses to dietary molybdenum challenge are summarized in Table 2.

**Table 2:** Species-specific growth and copper-status responses to dietary molybdenum challenge.

Species	Mo level	Exposure context/duration	Growth or production response	Copper-status response	Ref
Boer-cross goat	5–10 ppm	85 days	No significant ADG or final BW depression	Linear decrease in liver Cu	(24)
Calf/feedlot steer	Supplemental Mo + S	Extended feeding period	Potential growth depression under prolonged antagonism	Reduced plasma/liver Cu	(24, 29, 31)
Sheep	5–10 ppm/chronic exposure	Chronic exposure	Reduced weight gain and feed conversion efficiency	Severe Cu depletion; high toxicity risk if Cu is excessive	(12, 27, 28)
Dairy cow	High dietary or water Mo/S exposure	Lactation	Variable production response	Reduced Cu availability/status	(29, 31)

## Physiological and pathological consequences of molybdenum excess

Molybdenum does not merely act as an antagonist to copper; at high concentrations, it induces a state of multi-organ toxicity that disrupts core metabolic pathways (8, 32). Recent metabolomics research in goats has shed light on the systematic damage caused by chronic molybdenum exposure (50 mg/kg), particularly focusing on the pancreas and liver (8).

## Pancreatic dysfunction and oxidative stress

The goat pancreas is a major target for molybdenum-induced injury. Metabolomics analysis of pancreatic tissue has identified 167 differentially expressed metabolites (DEMs) in response to high Mo intake (8). These changes are concentrated in pathways critical for antioxidant defense, specifically the metabolism of glycine, serine, threonine, cysteine, and methionine (8, 33). Cysteine and methionine are essential precursors for the synthesis of glutathione, the primary cellular antioxidant (34, 35). The disruption of these pathways results in a marked reduction in total antioxidant capacity (T-AOC) and the activity of key enzymes such as superoxide dismutase (SOD), catalase (CAT), and glutathione peroxidase (GSH-Px) (8, 34, 36). Simultaneously, molybdenum induces a pro-inflammatory environment in the pancreas, evidenced by significant increases in interleukin-2 (IL-2), IL-6, and tumor necrosis factor-alpha (TNF-*α*) (8, 37). This combination of oxidative stress and inflammation leads to morphological damage, including cellular swelling and necrosis, which can impair the endocrine and exocrine functions of the pancreas, further complicating nutrient utilization and growth (8, 38, 39).

## Impact on immune competence

The relationship between copper status and immunity is well-established, with copper-deficient animals exhibiting increased susceptibility to infections (40–42). In goats, even when growth performance remains stable, additional molybdenum has been associated with linearly reduced immune responses in an 85-day experimental study, including lower humoral response to ovalbumin and lower cell-mediated response to phytohemagglutinin (24). This is reflected in significant decreases in both humoral and cell-mediated responses:

- *Ovalbumin response*: Humoral immune response decreased linearly with increasing dietary Mo, with a reported treatment effect of *p* = 0.001 in Boer-cross goats (24).
- *Phytohemagglutinin (PHA) response*: Cell-mediated immunity, assessed by the PHA skin-swelling response, also decreased linearly with increasing dietary Mo, with a reported treatment effect of *p* = 0.002 (24, 43).

These findings suggest that the “copper cost of immunity” is high; the animal prioritizes maintenance of core metabolic functions over immune surveillance when copper availability is restricted by molybdenum (24, 44). Consequently, ruminants in high-molybdenum environments are more prone to high parasite loads and infectious diseases (24, 32).

## Effects on rumen digestibility and microbial dynamics

The rumen is a highly coordinated fermentation chamber where microbial populations convert lignocellulose into volatile fatty acids (VFAs) and microbial protein (45, 46). The integrated ruminal, systemic, diagnostic, and mitigation pathways involved in Cu–Mo–S antagonism are summarized in Figure 1.

**Figure 1:**
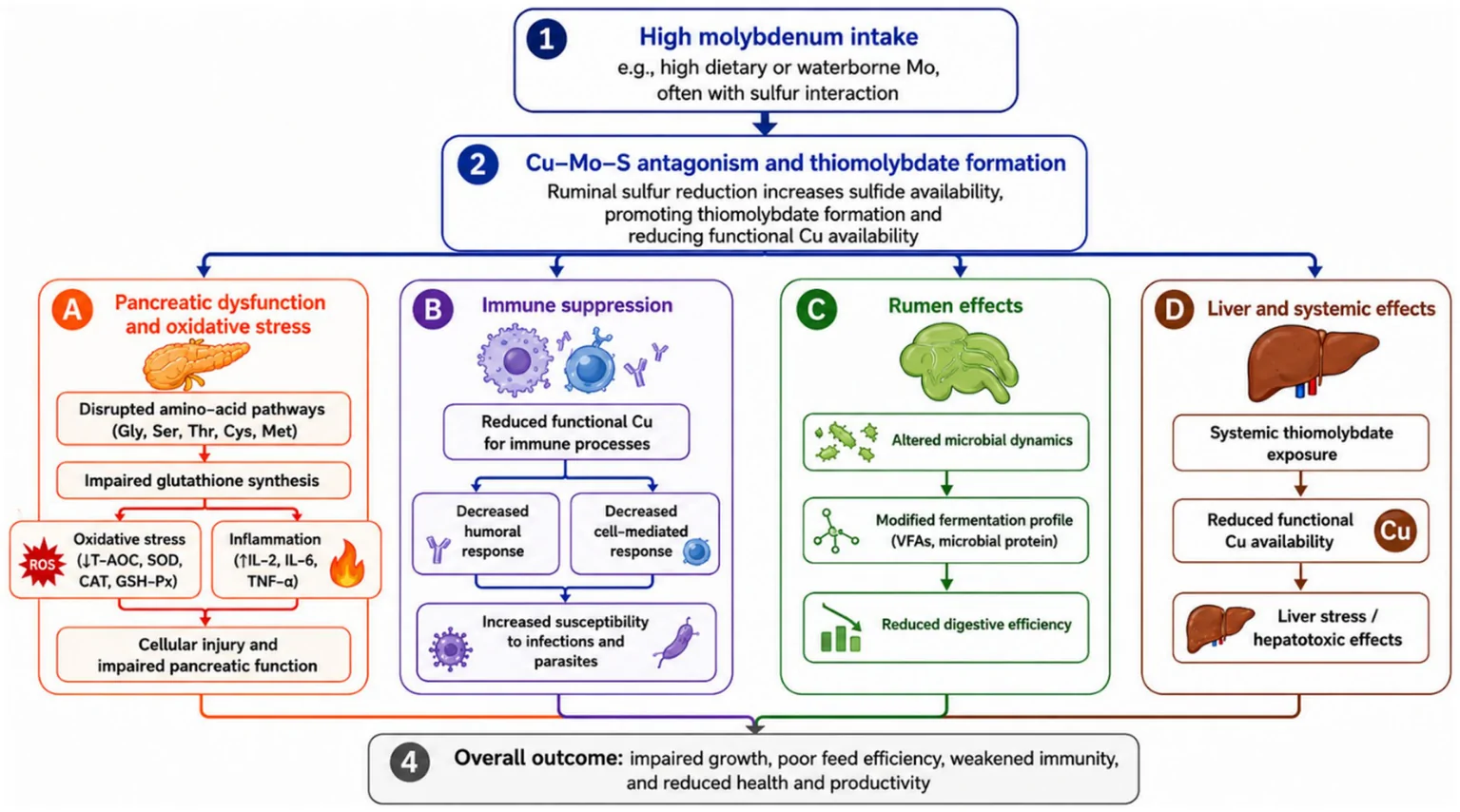
Integrated framework of Cu–Mo–S antagonism in ruminants. Dietary and water-derived copper, molybdenum, sulfur, iron, and zinc enter the rumen, where sulfur reduction increases sulfide availability and promotes thiomolybdate formation. In the presence of sufficient rumen-soluble copper, thiomolybdates form insoluble Cu–Mo–S complexes that are excreted. When thiomolybdate production exceeds rumen-available copper, thiomolybdates may be absorbed and bind systemic copper pools, reducing functional copper availability and interfering with copper-dependent enzymes. Clinical consequences include impaired growth, immune suppression, altered rumen fermentation, coat depigmentation, and reduced wool/fiber quality. Diagnostic and mitigation strategies include integrated feed-water mineral analysis, interpretation of Cu:Mo:S balance, serum and liver copper assessment, functional biomarkers, TCA-fractionated plasma copper where available, reduction of dietary antagonists, and cautious species-specific copper supplementation.

Beyond protozoal responses, recent multi-omics studies indicate that host performance and rumen fermentation are linked to shifts in microbial functional pathways, including sulfur metabolism and hydrogen utilization (17–19, 47–52).

## Microbial populations and ciliates

Molybdenum is an essential cofactor for certain bacterial enzymes, such as nitrate reductase, and at low levels, it can stimulate the digestion of cellulose and the utilization of sulfites by rumen microflora (53, 54). However, high concentrations are toxic to specific microbial groups. For instance, chronic high Mo intake significantly reduces the total number and vitality of ciliate protozoa in the rumen fluid (53, 55). Since ciliates play a key role in stabilizing fermentation and providing high-quality protein to the host, their reduction can disrupt the overall nutrient balance and decrease the efficiency of feed conversion (8, 56).

## Volatile fatty acid production and nutrient absorption

The interaction of copper and molybdenum also alters the fermentation patterns within the rumen. Studies have shown that the source and concentration of trace minerals can impact the molar proportions of VFAs (11, 19). In steers, sulfate sources of trace minerals (which may increase ruminal sulfide) have been associated with lower molar proportions of propionate compared to hydroxychloride sources (19, 57, 58). Given that propionate is the primary gluconeogenic precursor in ruminants, a shift away from its production can negatively impact energy availability for growth and lactation (59, 60). Therefore, the available evidence should be interpreted as showing that trace-mineral source and ruminal solubility can shift fermentation patterns, whereas direct causal links between Cu–Mo antagonism and specific VFA profiles still require controlled studies across diet types and species (19, 57, 58).

## Follicle characteristics and the biology of fiber quality

Perhaps the most diagnostic and visually evident consequence of copper-molybdenum imbalances is the alteration of hair and wool follicle function (61). Copper is fundamentally required for two critical processes in the follicle bulb: keratinization and pigmentation (9, 62).

## Keratinization and “steely wool”

The mechanical strength and physical properties of wool are derived from the cross-linking of keratin intermediate filaments (KIFs) with keratin-associated proteins (KAPs) (63, 64). This cross-linking requires the oxidation of thiol groups (-SH) on cysteine residues to form disulfide bonds (-S-S-) (9, 10). This biochemical transition is catalyzed by a copper-dependent enzyme, likely a thiol oxidase. In copper-deficient sheep—whether due to low dietary Cu or high Mo antagonism—this enzyme activity is compromised, leading to a failure in the formation of disulfide bridges (9, 65).

The resulting wool is referred to as “steely” or “stringy” wool. It is characterized by:

- *Loss of crimp*: The natural wave or crimp of the wool fiber disappears, resulting in a straight, hair-like appearance (9).
- *Reduced tensile strength*: The lack of cross-linking makes the fibers weak and prone to breakage during processing (9).
- *Increased lustre*: The fiber takes on a greasy, lustrous appearance that is undesirable in fine-wool production (9).

## Pigmentation and tyrosinase pathway

The pigmentation of the hair and wool coat is governed by the synthesis of melanin, a process initiated by the enzyme tyrosinase (66, 67). Tyrosinase is a copper-containing metalloenzyme that catalyzes the conversion of tyrosine to dihydroxyphenylalanine (DOPA) and subsequently to dopaquinone (9, 68). When systemic thiomolybdates sequester copper or when copper availability is low, tyrosinase activity drops precipitously (9, 69).

This leads to the characteristic depigmentation of the coat, often observed as:

- *Spectacle eye*: A grayish-brown discoloration or loss of hair around the margins of the eyes (12).
- *Banding in wool*: In black-wooled sheep, periods of copper deficiency are marked by bands of white or unpigmented wool alternating with pigmented sections, providing a chronological record of the animal’s mineral status (70).
- *Achromotrichia*: A general fading of the coat color, most noticeable in black or dark-colored cattle and goats (70) (Table 3).

**Table 3:** Follicle parameters and role of copper and molybdenum on it.

Follicle parameter	Role of copper	Consequence of Mo antagonism	Ref
Disulfide bridge formation	Thiol oxidase cofactor	Steely wool, loss of crimp	(9)
Melanin synthesis	Tyrosinase cofactor	Achromotrichia, “spectacle eye”	(9)
KAP expression	Structural matrix protein	Weakened fiber, thinned diameter	(9)
Collagen stability	Lysyl oxidase cofactor	Skin fragility, follicle detachment	(9)

## Diagnostic paradigms: from serum copper to molecular markers

A major hurdle in managing the Cu-Mo-S interaction is the lack of sensitive and specific biomarkers that can distinguish between simple deficiency, induced deficiency, and thiomolybdate toxicity (71, 72). Traditional measures, such as serum copper and liver copper concentrations, have significant limitations in the presence of molybdenum (73, 74).

## Limitations of current indicators

Serum copper reflects the transport pool but does not necessarily correlate with cellular function or tissue stores, especially when thiomolybdates are present (22, 73). As previously discussed, thiomolybdates can increase total serum copper by creating a TCA-insoluble pool that is metabolically inert (12). Liver copper is a better indicator of total reserves, but it is an invasive measure and may not reflect the immediate metabolic challenges faced by peripheral tissues like the follicles or the immune system (75, 76).

## Emerging molecular biomarkers

The search for “early warning” signs of copper imbalance has led to the identification of several molecular candidates.

## Copper chaperone for superoxide dismutase

CCS is perhaps the most promising novel biomarker for copper status. Its primary role is to deliver copper to the enzyme superoxide dismutase 1 (SOD1) (77, 78). Research has shown that CCS protein levels are inversely related to copper status; in copper-deficient beef cattle, CCS protein levels increased by 40% in the liver and 65% in erythrocytes (77, 79, 80). Crucially, unlike the acute-phase protein ceruloplasmin, CCS protein levels are not affected by inflammation or vaccination, making it a highly reliable indicator of functional copper status (77, 79).

## Hepatic mRNA expression

Transcriptomic analysis of bovine liver tissue has revealed that several genes involved in copper homeostasis are regulated by copper concentration (81). Specifically, the expression of the copper transporter protein (CTR1), and the copper-transporting ATPases (ATP7A and ATP7B), are negatively correlated with hepatic copper status (82, 83). As copper stores are depleted, the expression of these transporters is upregulated to maximize uptake and internal trafficking (82, 84).

## MicroRNA (miRNA) profiling

High-throughput sequencing has identified miRNAs as promising regulatory molecules and non-invasive biomarkers in livestock health, nutrition, reproduction, and metabolic stress (85–87). However, direct evidence linking specific miRNA signatures to Cu–Mo–S antagonism in ruminants remains limited. Plant studies have identified Cu-responsive miRNAs, such as miR397 and miR398, that participate in copper allocation under deficiency conditions (85, 86, 88–91), but these mechanisms should not be directly extrapolated to ruminants. In ruminant research, circulating and extracellular-vesicle miRNAs are better framed as exploratory biomarkers that may eventually complement mineral-status indicators, rather than as validated diagnostic tools for thiomolybdate-induced copper dysfunction (85–87). Future studies should therefore test whether Cu deficiency, high Mo/S exposure, or confirmed thiomolybdate challenge produces reproducible miRNA profiles in blood, liver, follicular tissue, or reproductive tissues.

## Precision supplementation and individualized care

The traditional practice of providing free-choice mineral mixes to a herd assumes that all animals will consume enough to meet their requirements while avoiding toxicity. However, research using electronic feeders and precision monitoring systems has debunked this assumption (87). Copper supplementation in cattle is clinically challenging because the Cu–Mo–S interaction can induce secondary deficiency, whereas excessive liver copper accumulation may increase toxicity risk when copper is supplied above requirements (92).

## The SmartFeed and MCCC paradigms

Technologies such as the SmartFeed system (C-Lock Inc.) and the Mobile Cow Command Center (MCCC) allow for the precise measurement of individual mineral intake in grazing cattle (93). These systems utilize RFID technology to identify each animal and record the amount and duration of feeding.

Data from grazing Angus cow-calf pairs showed that mineral intake is highly variable:

- *Cows*: High-intake individuals averaged 125.4 g/d, while low-intake individuals averaged only 33.5 g/d (93).
- *Calves*: Consumption was even more erratic, with high-intake calves averaging 72.2 g/d and low-intake calves at 22.2 g/d (93).

This variability has direct physiological consequences. Cows with high mineral intake, as recorded by the SmartFeed system, had significantly greater liver concentrations of copper (247 vs. 116 μg/g) and selenium (93). This suggests that “average” herd supplementation often leaves a significant portion of the population at risk of deficiency, while others may approach toxic thresholds (93).

## Strategic use of rumen-protected minerals

To circumvent the Cu-Mo-S interaction in the rumen, there is an increasing focus on the use of organic or rumen-protected mineral sources. Copper-amino acid complexes and hydroxychlorides are designed to remain stable at the ruminal pH, preventing the formation of insoluble copper-thiomolybdate complexes (12, 94). While some trials have shown limited differences in overall nutrient digestibility, organic sources have been shown to maintain higher ceruloplasmin activity and serum copper concentrations when combined with organic sulfur sources in lambs (94, 95). This suggests that the bio-pathway of organic minerals is distinct and less prone to sequestration by thiomolybdates (94).

## Practical diagnostic and intervention framework for Cu–Mo–S imbalance

From a clinical and herd-management perspective, Cu–Mo–S imbalance should be approached as a risk-stratification problem rather than as a diagnosis based on a single copper measurement. The first step is to analyze the total mixed ration, forage, mineral supplement, and drinking water for Cu, Mo, S, Fe, and Zn. Particular attention should be given to diets containing both elevated Mo and S, because diets with Mo > 5 mg/kg DM and S > 0.33% have been reported to reduce Cu status in cattle and sheep (29, 96). In cattle, the basal Cu requirement is commonly around 10 mg/kg DM, but antagonistic pressure from Mo and S may increase supplementation requirements (25, 29, 96). In sheep, intervention must be more conservative because sheep are highly susceptible to chronic copper accumulation and toxicity; dietary Cu concentrations of approximately 5–10 mg/kg DM are generally considered adequate under normal Mo and S conditions, whereas excessive Cu exposure can be hazardous (18, 28).

A practical diagnostic algorithm is proposed as follows. First, if clinical signs such as ill-thrift, coat depigmentation, diarrhea, impaired immunity, reduced fertility, or poor fiber quality are present, dietary and water sources should be tested for Cu, Mo, S, Fe, and Zn. Second, the dietary Cu: Mo ratio and total sulfur load should be interpreted together rather than separately. A low Cu: Mo ratio, especially in the presence of high sulfur, supports secondary Cu deficiency or thiomolybdate challenge. Third, animal-level testing should include serum Cu, ceruloplasmin activity, liver Cu where feasible, and functional indicators such as erythrocyte SOD1 or CCS when available. Fourth, if serum Cu is normal or high but clinical signs and dietary antagonists are present, thiomolybdate-induced functional Cu deficiency should be suspected rather than excluded. In such cases, TCA-fractionated plasma Cu may help distinguish metabolically available copper from TCA-insoluble thiomolybdate–copper–protein complexes (12). Finally, intervention should prioritize removal or reduction of antagonists, correction of the total dietary Cu: Mo: S balance, and species-specific supplementation under veterinary supervision, with particular caution in sheep because of the narrow safety margin between deficiency and toxicity (28, 29, 96). A simplified diagnostic framework for field and laboratory decision-making is provided in Table 4.

**Table 4:** Practical diagnostic framework for differentiating dietary Cu deficiency from thiomolybdate-induced functional Cu deficiency.

Step	Assessment	Suggests primary dietary Cu deficiency	Suggests thiomolybdate-induced functional Cu deficiency	Practical response
1	Diet/water mineral analysis	Low dietary Cu with normal Mo and S	Adequate Cu with high Mo and/or S; low Cu: Mo ratio	Analyze forage, TMR, mineral mix, and water
2	Clinical pattern	General hypocuprosis signs	Hypocuprosis signs despite apparently adequate Cu intake	Check antagonists before increasing Cu
3	Serum Cu	Usually low	Low, normal, or occasionally misleadingly normal/high	Do not rely on serum Cu alone
4	Liver Cu	Low reserves	Variable; may not reflect immediate functional blockade	Use as reserve indicator, not sole diagnostic marker
5	Ceruloplasmin/SOD1/CCS	Reduced Cu-dependent function	Functional Cu impairment likely	Add functional biomarkers where available
6	TCA-fractionated plasma Cu	Low available Cu without TCA-insoluble complex dominance	Increased TCA-insoluble thiomolybdate–Cu–protein complex	Supports systemic thiomolybdate exposure
7	Intervention	Increase Cu to species-appropriate level	Reduce Mo/S antagonism, adjust Cu:Mo:S balance, consider protected Cu	Avoid aggressive Cu supplementation in sheep

## Future directions: multi-omics and genomic resilience

As we look toward the next decade of ruminant nutrition, the integration of multi-omics data will be central to resolving the complexities of mineral interactions.

## Genomic selection for mineral efficiency

Ruminant species and breeds exhibit significant differences in their “tolerance” of copper and molybdenum (24, 40). Sheep, for instance, have a limited capacity to synthesize metallothionein (MT) compared to pigs, which contributes to their sensitivity to copper handling (28, 40). Future research aims to identify the genomic signatures associated with efficient copper transport and resistance to thiomolybdate challenge. The use of telomere-to-telomere (T2T) gap-free genome assemblies and graphical pangenomics will facilitate the identification of functional variants that can be incorporated into elite breeding programs to produce livestock better adapted to high-molybdenum environments (97).

## Maternal programming and the epigenome

Maternal nutrition during gestation is a powerful driver of offspring development. Multi-omics analyses of beef cattle offspring have shown that gestational mineral and protein supplementation induces long-term changes in the muscle transcriptome, plasma metabolome, and ruminal microbiome (98, 99). These “programming” effects suggest that managing the copper-molybdenum balance in the dam is not only critical for her own health but is essential for setting the metabolic and immune trajectory of the calf (98, 99). Future studies will likely explore the epigenetic mechanisms, such as DNA methylation and histone modification, that underpin these long-term responses to prenatal mineral status.

## Conclusion

Evaluating the influence of copper and molybdenum on ruminant health reveals a sophisticated biochemical struggle centered in the rumen but manifesting in every organ system. The synthesis of thiomolybdates remains the primary mechanism of antagonism, causing a shift in copper availability that traditional diagnostic tools often fail to capture. The resulting functional deficiency has been shown to linearly depress immune function and permanently alter the structural and aesthetic properties of hair and wool follicles through the inhibition of thiol oxidase and tyrosinase. Together, precision monitoring and functional biomarkers provide a practical framework for detecting mineral imbalance earlier and tailoring supplementation more safely across individuals. However, because the present review did not perform pooled quantitative synthesis, these conclusions should be interpreted as mechanistic and evidence-mapping inferences rather than meta-analytic estimates of effect size. No longer can we rely on group-average intake; the variability between individuals necessitates technology-driven, individualized care. Furthermore, the integration of multi-omics approaches in 2024 and 2025 provides a holistic view of how mineral status influences the animal from the level of the ruminal microbiome to the expression of the host’s genome. By harnessing these current insights from the chemistry of the rumen to the transcriptomics of the follicle, we can develop more resilient production systems that mitigate the risks of molybdenum excess and optimize the life-long productivity and welfare of ruminant livestock. Future research must continue to prioritize the translation of these molecular findings into practical, field-deployable diagnostic kits and precision supplementation strategies to ensure the sustainable intensification of global animal agriculture.
